# Plasma concentration and eGFR in preterm and term neonates receiving gentamicin or successive amikacin therapy

**DOI:** 10.1186/s12887-023-03834-4

**Published:** 2023-01-16

**Authors:** Bonifasius Siyuka Singu, Milka Ndapandula Ndeunyema, Ene I. Ette, Clarissa Hildegard Pieper, Roger Karel Verbeeck

**Affiliations:** 1 School of Pharmacy, Faculty of Health Sciences, University of Namibia, Private Bag, Windhoek, 13301 Namibia; 2 Neonatal Unit, Windhoek Central Hospital, Windhoek, Namibia

**Keywords:** Neonates, Kidney function, Gentamicin, Amikacin, Nephrotoxicity

## Abstract

**Background:**

Gentamicin and amikacin are aminoglycoside antibiotics which are renally excreted and known to be nephrotoxic. Estimate of glomerular filtration rate (eGFR) per body surface area is lower in neonates than in adults and exposure to these drugs could lead to more suppression in kidney function. The aim of this study was to determine maximum and minimum plasma concentrations (C_max_ and C_min_), time to reach C_min_ levels of gentamicin and amikacin, and to assess eGFR in preterm and term neonates.

**Methods:**

Two groups of patients were recruited, 44 neonates receiving gentamicin (5 mg/kg/24 h) and 35 neonates receiving amikacin (15 mg/kg/24 h) by slow intravenous injection. Patients on amikacin had been on gentamicin before being switched to amikacin. Two blood samples were drawn for the determination of the maximum and minimum plasma concentration. Primary outcomes were determination of C_max_, C_min_, and the time it took to clear the aminoglycoside to a plasma concentration below the toxicity threshold (gentamicin: < 1 mcg/mL; amikacin: < 5 mcg/mL.

**Results:**

Therapeutic range for C_max_ of gentamicin (15–25 mcg/mL) or amikacin (30–40 mcg/mL) was achieved in only 27.3 and 2.9% of neonates, respectively. Percentage of neonates reaching plasma concentrations below the toxicity threshold within the 24-hour dosing interval was 72.7% for gentamicin and 97.1% for amikacin. Positive correlation between gentamicin clearance and postnatal age borderline statistical significance (*p* = 0.007), while the correlation between amikacin clearance and postnatal age was poor and not statistically significant (r^2^ = − 0.30, *p* = 0.971).

**Conclusion:**

Although eGFR decreased significantly as a function of postnatal age in neonates receiving amikacin, the majority (91.4%) of these neonates were able to clear the drug to < 5 mcg/mL within a 24-hour dosing interval.

## Key points

The practice of successive administration of gentamicin and amikacin as first- and second-line therapy, respectively, increases chances of kidney function suppression in neonates due to prolonged exposure to these nephrotoxic drugs. Although eGFR decreased significantly as a function of postnatal age in neonates receiving amikacin, almost all (97.1%) of these neonates were able to clear the drug to < 5 mcg/mL within a 24-hour dosing interval.

## Introduction

Gentamicin and amikacin are aminoglycoside antibiotics that have concentration-dependent bactericidal action and are primarily used against infections due to aerobic gram-negative bacteria [1]. However, these potent broad-spectrum antimicrobials should be used with caution due to the associated drug-induced adverse effects, namely, nephrotoxicity and ototoxicity which result from accumulation and retention of the drug in the proximal tubular cells of the kidneys, and the labyrinth of the ear, respectively [1, 2]. Over 90% of the aminoglycoside dose is eliminated by excretion through the kidneys [1, 3]. Nephrotoxicity due to aminoglycosides correlates with total dose and duration of therapy and is known to result in reversible mild renal impairment in 8–26% of patients characterized by a rise in serum creatinine concentrations; the condition is reversible because proximal tubular cells have the capacity to regenerate [4, 5]. In comparison to adults, neonates have a consistently lower rate of oto- and nephrotoxicity due to maturation-related low-expression of megalin and cubulin which are ligands that facilitate the uptake of aminoglycosides at the surface of cochlear hair and renal tubular cells [6]. The World Health Organization (WHO) recommends gentamicin in combination with ampicillin or penicillin as first-line therapy against neonatal sepsis for synergy and because of low reported resistance patterns to gentamicin [7–9]. Therapeutic drug monitoring (TDM) is used to limit the occurrence of toxicity in neonates undergoing aminoglycoside therapy [9, 10]. For once-daily dosing, minimum or trough concentrations (C_min_) for gentamicin are recommended to be < 1 mcg/mL and 15–25 μg/mL for maximum or peak concentrations (C_max_), while for amikacin the troughs should be < 5 mcg/mL and 30–40 mcg/mL for peak concentrations [10–15]. The ratio between the peak concentration and the minimal inhibitory concentration (Cmax/MIC) for aminoglycoside antibiotics is considered to be 8–10 [16]. In neonates, the pharmacokinetic approach to dosage adjustment is preferred to the high-dose extended interval method [1, 14, 17–19]. Although the nephrotoxic effect of gentamicin and amikacin in neonates is well documented, studies involving neonates receiving gentamicin as first-line followed by amikacin as second-line therapy to report on the effect of such successive therapy on neonatal renal function are lacking.

The aim of this study was to determine the peak and trough plasma concentrations of gentamicin and amikacin, the time to reach the recommended trough concentrations (T_min_), and to assess eGFR in preterm and term neonates.

## Methods

### Study setting and patient population

This cross-sectional clinical study was conducted at the Neonatal Unit of the Maternity Ward at the Windhoek Central Hospital in Windhoek, Namibia. Plasma concentrations of gentamicin and amikacin were derived from two separate population pharmacokinetic studies in which one cohort of neonates were receiving gentamicin and the other group were receiving amikacin in the same neonatal unit using the same methodology. Data for gentamicin were collected from 44 neonates starting from 4 July 2019 to 29 December 2019, while data for amikacin were collected from 35 neonates from 25 July 2020 to 7 December 2020 (all data were collected over a total period of 1 year). The cohort of neonates who received gentamicin was not the same as the cohort of neonates who received amikacin, but all neonates who received amikacin had first been treated with gentamicin as first-line treatment of sepsis. Patients who were included in the study were those who had two reported drug concentrations, and at least one serum creatinine measurement.

### Study procedure

Aminoglycoside therapy was prescribed against either suspected or confirmed sepsis. Gentamicin in combination with a penicillin such as penicillin G or ampicillin is indicated as first-line in prophylaxis against suspected neonatal sepsis where there are risk factors such as premature birth, very low (and extremely low) birthweight (VLBW and ELBW), and respiratory distress syndrome (RDS). Amikacin was given in combination with piperacillin/tazobactam as second-line therapy if signs of sepsis persisted even after the gentamicin course had been completed.

Gentamicin therapy was initiated soon after birth, while treatment with amikacin was only initiated when the gentamicin course had been completed with no improvement in the patient’s condition.

The aminoglycoside dose, 5 mg/kg/24 h for gentamicin and 15 mg/kg/24 h for amikacin (both courses prescribed to be administered for 5 or 7 days as per hospital guidelines), was administered by the ward nurses as a slow intravenous injection (slow bolus injection) over 3–5 seconds via a cannula and two blood samples were drawn by venipuncture thereafter. Each blood sample was taken at a time falling in either of the following sampling blocks: 4–6 minutes (0.06–0.10 h), 6–180 minutes (0.1–3.0 h), or 180–320 minutes (3–5.4 h) after the initial or successive doses. Blood samples were collected into sterile 500 μL serum separating tubes, centrifuged and serum was stored frozen in Eppendorf tubes at − 20 °C until analysed.

### Laboratory methods

Serum creatinine concentrations were measured by the kinetic alkaline pic rate Jaffe method using the Cobas® 6000 analyzer (Roche Diagnostics, IN, USA). Gentamicin and amikacin concentrations were determined using the Indiko Plus™ autoanalyzer (Thermo Fisher Scientific Inc., CA, USA). The lower limits of quantification for gentamicin and amikacin were stated by the manufacturer as being 0.3 μg/mL and 0.8 μg/mL, respectively.

### Data analysis

The two reported drug concentrations were plotted onto semi-logarithmic graph paper to estimate the half-life, C_max_, and the time to reach the target trough concentration which was < 1 mcg/mL for both gentamicin and amikacin. The eGFR was estimated by using the Schwartz method for estimating creatinine clearance in neonates (eq. 1) [16]:

1 $$\textrm{eGRF}\left(\textrm{in}\ \textrm{mL}/\min /1.73\ {\textrm{m}}^2\right)=\frac{\textrm{Factor}\times \textrm{Height}\ \left(\textrm{in}\ \textrm{cm}\right)}{\textrm{Serum}\ \textrm{creatinine}\ \left(\textrm{in}\ \textrm{mg}/\textrm{dL}\right)}$$

where: Factor is 0.43 in term neonates or 0.33 in preterm neonates.

The results were summarized using descriptive statistics such as median, range, and percentages in R (R version 3.6.2). The Mann-Whitney U test was used to compare means, a *p*-value < 0.05 was considered statistically significant.

## Results

**Fig. 1:**
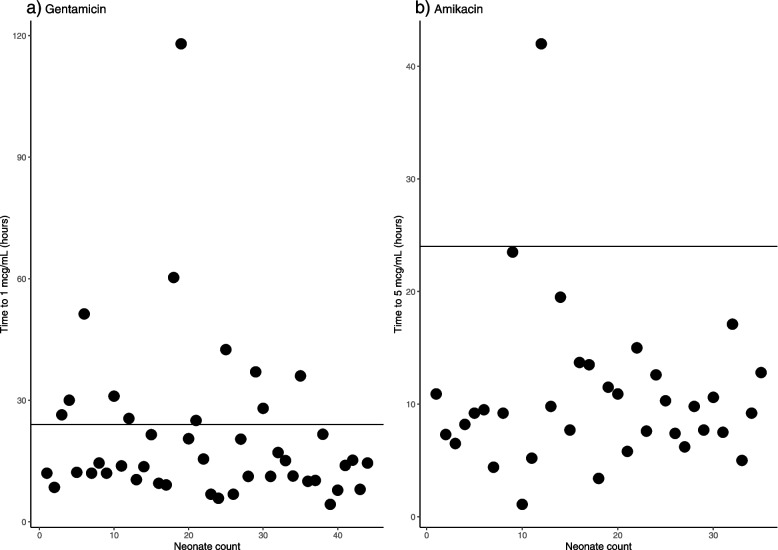
The time it took for aminoglycoside concentrations to reach the recommended Trough concentrations, the horizontal line marks 24 hours. **a** gentamicin: *n* = 44, Trough < 1 mcg/mL), and **b** amikacin: *n* = 35, Trough < 5 mcg/mL

**Fig. 2:**
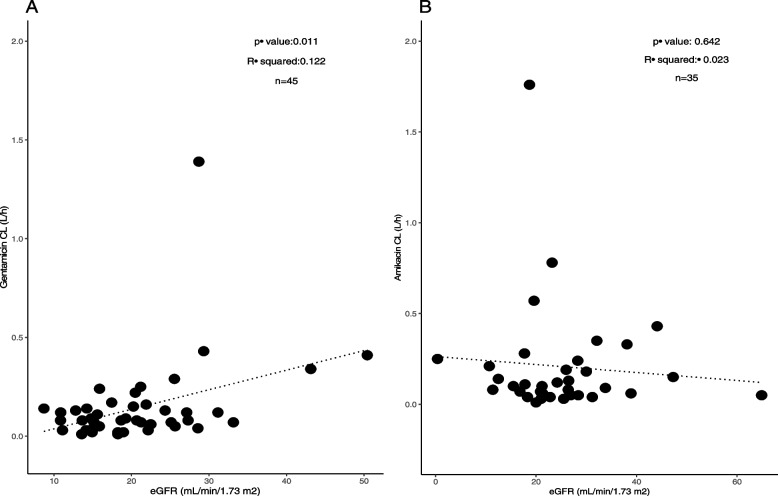
Correlations of clearance for gentamicin (in **A**) and amikacin (in **B**) with eGFR

**Fig. 3:**
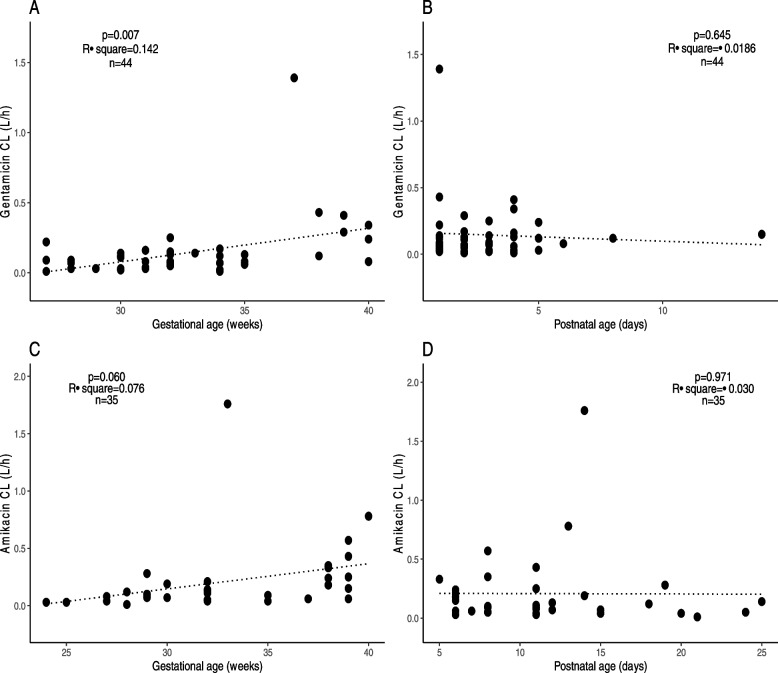
Correlation of CL with gestational age (**A** and **C**), postnatal age (**B** and **D**), in neonates while receiving gentamicin and amikacin. In D, a negative correlation can be observed between CL and postnatal age in newborns while receiving amikacin and had previously been exposed to gentamicin. Exclusion of outliers (gentamicin: one neonate with a PNA of 14 days; amikacin: two neonates with eGFR values of 0.4 and 64.9 mL/min/1.73 m^2^) does not produce statistically significant in both correlations of CL vs PNA

Of the 44 neonates on gentamicin and 35 who received amikacin, 81.8 and 68.6% were of premature birth, respectively. The two groups of neonates had very similar demographic characteristics with no statistical difference: gestational age (GA) (W = 753, *p* = 0.870), height (W = 706, *p* = 0.527), and birth weight (W = 862, *p* = 0.367) (Table 1). Gentamicin (5 mg/kg/24 h) and amikacin (15 mg/kg/24 h) were given for a median duration of 5 and 7 days, respectively (Table 1). Gentamicin was initiated at a median PNA of 3 days (range: 1–14 days), and amikacin at 12 days (range: 5–25 days). Target peak concentrations were better achieved for gentamicin compared with amikacin (Table 2); peak concentrations fell within the therapeutic range in 27.3% of the neonates treated with gentamicin versus 2.9% for the amikacin cohort. In 72.7 and 97.1% of the study subjects the toxicity threshold (or target trough) was reached within 24 hours for gentamicin (< 1 mcg/mL) and amikacin (< 5 mcg/mL), respectively (Fig. 1). Although the *p*-values were close to significance, there was no statistical difference in the eGFR between the two cohorts (*p* = 0.060), or when only the preterm babies from the cohorts were compared (*p* = 0.068). The correlation between gentamicin clearance and eGFR was statistically significant (*p* = 0.011, *r*^2^ = 0.122) but not for amikacin clearance with eGFR (*p* = 0.642, *r*^2^ = − 0.023) (Fig. 2). Correlation of clearance with GA was positive and statistically significant for gentamicin (*p* = 0.007, *r*^2^ = 0.142) but not for amikacin (*p* = 0.060, *r*^2^ = 0.076). However, correlations between gentamicin and amikacin clearance with PNA were both weakly negative and not statistically significant: gentamicin (*p* = 0.642, *r*^2^ = 0.075), and amikacin (*p* = 0.971, *r*^2^ = − 0.030) (see Fig. 3). Where data on mortality were available, 6 out of 43 (14.0%) neonates on gentamicin died while still receiving gentamicin therapy, and 3 out 26 (11.5%) died during amikacin therapy.

**Table 1:** Patient demographics and pharmacokinetic parameters for neonates on gentamicin and amikacin

		Gentamicin (*n* = 44)	Amikacin (*n* = 35)
**Sex**	Male	25 (56.8%)	23 (65.7%)
	Female	19 (43.2%)	12 (34.3%)
**Status**	Preterm (< 37 weeks)	36 (81.8%)	24 (68.6%)
	Term	8 (18.2%)	11 (31.4%)
**Gestational age (weeks)**	Median (range)	32 (27–40)	32 (24–40)
**Postnatal age (days)**	Median (range)	3 (1-14)	12 (5–25)
**Postmenstrual age (days)**	Median (range)	227 (190–285)	236 (179–293)
**Birth weight**^a^	Median (range), (g)	1573 (895–3920)	1920 (940–4500)
	NBW (≥ 2500 g)	8 (18.2%)	9 (25.7%)
	LBW (< 2500 g)	19 (43.2%)	14 (40.0%)
	VLBW (< 1500 g)	15 (34.1)	10 (28.6%)
	ELBW (1000 g)	2 (4.5%)	2 (5.7%)
**Birth height (cm)**	Median (range)	42 (30–53)	41 (28–55)
**Body Surface Area (m**^**2**^**)**	Median (range)	0.137 (0.088–0.240)	0.149 (0.088–0.267)
**eGFR (mL/min/1.73 m**^**2**^**)**	Median (range)	20.3 (8.7–50.4)	23.2 (10.7–64.9)
**Dose (mg)**	Median (range)	8.0 (4.5–17.0)	29 (14–62)
**Peak concentration or C**_**max**_ **(mcg/mL)**	Median (range)	16 (4–64)	68 (11–260)
**Half-life (h)**		3.8 (1.4–28.0)	2.4 (0.8–9.5)
**Drug clearance (L/h)**		0.08 (0.01–1.39)	0.10 (0.01–1.76)
**Time to reach toxicity threshold (gentamicin: 1 mcg/mL; amikacin: 5 mcg/mL) (h)**	Median (range)	14.2 (4.3–118.0)	9.2 (1.1–42.0)
**Duration of therapy (days)**	Median (range)	5 (2–8)	7 (3–12)

^a^*NBW* Normal body weight*, LBW* Low body weight*, VLBW* Very low body weight*, ELBW* Extremely low body weight

**Table 2:** Proportion of neonates with Peak drug concentrations falling within the recommended ranges

	Peak concentration (C_**max**_)	Proportion of patients
**Gentamicin** (*n* = 44)	Below target range: < 15 mcg/mL	40.9%
	Within target range: 15–25 mcg/mL	27.3%
	Above target range: > 25 mcg/mL	31.8%
**Amikacin** (*n* = 35)	Below target range: < 30 mcg/mL	8.6%
	Within target range: 30–40 mcg/mL	2.9%
	Above target range: > 40 mcg/mL	88.6%

## Discussion

Gentamicin and amikacin are efficacious antibiotics that are useful in the management of infectious diseases, but they are also known to be associated with two serious adverse reactions, namely, ototoxicity and nephrotoxicity [1]. The effects on neonatal renal function due to successive use of these two aminoglycosides as first- and second-line therapy in a clinical setting have not been reported. It is suspected that prolonged exposure to these drugs could lead to nephrotoxicity. Studying the impact of the current successive use of gentamicin and amikacin on the kidney function of newborns will inform on appropriate dosage regimens design which will ensure safety by minimizing nephrotoxicity and toxicity.

In this study, concentrations of gentamicin and amikacin in serum were measured to determine C_max_ values and time taken to reach C_min_. For Both gentamicin and amikacin, drug clearance had a positive correlation with GA. This is expected since neonates born after a longer gestation will have a more advanced renal function than those born after a shorter gestation period [20]. In addition, neonates on amikacin had a more developed renal capacity going into their second or third week of life and therefore were able to clear the drug to safe levels before the next dose in virtually all cases. This is the same reason why eGFR was higher in amikacin than in gentamicin treated neonates. A study of 91 neonates receiving 5–6 mg/kg/day of gentamicin reports that 63% had potentially toxic trough concentrations (> 1 mcg/mL) of whom most were premature with lower gestational age, low birthweight, and in their first week of life [21]. The results of the present study show that when eGFR is correlated with PNA, eGFR significantly declined while neonates were receiving amikacin. Initiation of gentamicin was as early as day 1 after birth and therapy lasted for 5 days. Amikacin therapy, however, was only initiated after gentamicin therapy had failed and therefore was started at a median PNA of 12 days. Kidney function declined in neonates receiving amikacin from the start of therapy. The lack of improvement of renal function with increasing neonatal chronological age observed in amikacin neonates could be due to the successive exposure of the newborns to the two aminoglycosides which resulted in nephrotoxicity. The impact of prolonged exposure to amikacin on postnatal serum creatinine dynamics in ELBW neonates has been reported [22]. However, it worth noting that in 89.6% of neonates receiving amikacin, piperacillin/tazobactam was also administered as a comedication. Piperacillin/tazobactam has been shown to cause elevation in serum creatinine concentrations because it interferes with the Jaffe serum creatinine method [23], which was used to measure creatinine levels for this study, and therefore may have resulted in the underestimation of eGFR Furthermore, piperacillin/tazobactam has been reported to be a potential risk factor for acute kidney injury which could have contributed to the decrease of amikacin clearance values [24]. These two reasons could have contributed to the under estimation of eGFR and decrease of clearance values in amikacin neonates. In addition, it has been shown that sepsis itself is associated with acute kidney injury or reduced renal function which may suggest that the decrease in eGFR with PNA could also be due to the disease, and not only the aminoglycoside therapy [25]. Achievement of recommended C_max_ values for both gentamicin and amikacin was not high (27.3 and 2.9%, respectively) and large proportions of patients had concentrations that were supratherapeutic (31.8% for gentamicin and 88.6% for amikacin). Increasing the mg/kg dose across the board might result in more patients with concentrations in the therapeutic range but will also lead to many more neonates with supratherapeutic concentrations which may increase the risk of nephrotoxicity. The best solution would be to individualize the dose according to each patient’s estimated aminoglycoside plasma clearance using eq. 2:

2 $$\textrm{Dose}=\textrm{target}\ {\textrm{C}}_{\textrm{max}}\times \textrm{CL}\times \uptau$$

where CL is the drug clearance, and *τ* is the dosing interval.

Several factors are known to affect renal function in neonates leading to altered drug exposure and response, namely, kidney development/maturation, underlying kidney diseases/comorbidities, medications, and environmental and genetic factors [20]. Nephrogenesis begins and concludes between weeks 5–35 of gestation followed by improved intrarenal blood flow after birth, but renal function in the neonate is still much lower in comparison to that of adults because of the following factors: immature glomerular filtration and tubular secretory mechanisms, reduced renal perfusion pressure, and insufficient osmotic load to produce full counter-current effects [20]. Because preterm neonates are born at < 37 weeks of gestation while nephrogenesis is still incomplete their capacity for renal excretion/elimination of drugs is the most affected [26]. A comparison between neonates born small for gestational age and preterm neonates with appropriate weight for gestational age showed that neonates with a lower body weight also have a reduced renal function [27]. Although it is difficult to ascertain the association of gentamicin and toxicity in neonates as poor feeding, asphyxia and sepsis are reported to also cause acute kidney injury [28–30], studies have shown that gentamicin and amikacin are potentially nephrotoxic and are known to cause acute kidney injury in neonates leading to impaired renal function [31, 32]. A study by Darmstadt et al. (2008) reports that the risk of toxicity due to gentamicin is increased by repeat courses, persistent exposure to trough concentrations above 2 μg/mL for more than 10 days and renal impairment as a pre-existing condition [33]. Impaired renal function results in decreased GFR which has a direct effect on the clearance of drugs that are eliminated primarily by glomerular filtration [34–36]. This leads to higher plasma concentrations, prolonged half-life and increased exposure to such drugs [21].

A limitation of this study is that the neonates who were on gentamicin were not the same patients as those on amikacin therapy, they were two different groups of neonates. Aminoglycosides exhibit concentration-dependent bactericidal effect, and therefore the Peak/MIC ratios are the relevant pharmacokinetic-pharmacodynamic (PK/PD) parameter. However, the lack of minimum inhibitory concentrations (MIC) data is another limitation of this study.

## Conclusions

The results of this study suggest that successive exposure of neonates to gentamicin and amikacin as first- and second-line therapy, respectively, could lead to nephrotoxicity as demonstrated by the significant decline in eGFR in patients who had been exposed to aminoglycoside therapy longer.

However, the proportions of patients who were able to clear the drug to reach concentrations below the toxicity threshold within the dosage interval of 24 hours were considerably high (72.7% for gentamicin, and 97.1% for amikacin). To improve efficacy and safety of gentamicin and amikacin in newborns, eGFR should be monitored and the dose can be adjusted using the pharmacokinetic approach.

## Data Availability

The datasets used and/or analysed during the current study available from the corresponding author on reasonable request.

## Abbreviations

C_max_: Maximum serum drug concentrations
C_min_: Minimum serum drug concentrations
Mcg: Microgram
CL: Drug clearance
eGFR: Estimate glomerular filtration rate
GA: Gestational age
PNA: Postnatal age
PMA: Postmenstrual age (i.e. GA + PNA)
TDM: Therapeutic drug monitoring
